# Safety and Feasibility of Functional Repetitive Neuromuscular Magnetic Stimulation of the Gluteal Muscles in Children and Adolescents with Bilateral Spastic Cerebral Palsy

**DOI:** 10.3390/children10111768

**Published:** 2023-10-31

**Authors:** Leonie Grosse, Julian F. Schnabel, Corinna Börner-Schröder, Malina A. Späh, Anne C. Meuche, Nico Sollmann, Ute Breuer, Birgit Warken, Matthias Hösl, Florian Heinen, Steffen Berweck, Sebastian A. Schröder, Michaela V. Bonfert

**Affiliations:** 1 Division of Pediatric Neurology and Developmental Medicine, Department of Pediatrics—Dr. von Hauner Children’s Hospital, LMU Hospital, Ludwig-Maximilians-Universität München, 80337 Munich, Germanysberweck@schoen-klinik.de (S.B.);; 2 LMU Center for Children with Medical Complexity—iSPZ Hauner, Ludwig-Maximilians-Universität München, 80337 Munich, Germany; 3 Department of Diagnostic and Interventional Neuroradiology, School of Medicine, Klinikum rechts der Isar, Technical University of Munich, 81675 Munich, Germany; nico.sollmann@tum.de; 4 TUM-Neuroimaging Center, Klinikum rechts der Isar, Technical University of Munich, 81675 Munich, Germany; 5 Department of Diagnostic and Interventional Radiology, University Hospital Ulm, 89081 Ulm, Germany; 6 Gait and Motion Analysis Laboratory, Schoen Clinic Vogtareuth, 83569 Vogtareuth, Germany; 7 Specialist Center for Pediatric Neurology, Neurorehabilitation and Epileptology, Schoen Clinic Vogtareuth, 83569 Vogtareuth, Germany

**Keywords:** neurostimulation, repetitive peripheral magnetic stimulation, motor impairment, physical exercise, selective motor control

## Abstract

**Background**: For children and adolescents affected by bilateral spastic cerebral palsy (BSCP), non-invasive neurostimulation with repetitive neuromuscular magnetic stimulation (rNMS) combined with physical exercises, conceptualized as functional rNMS (frNMS), represents a novel treatment approach. **Methods**: In this open-label study, six children and two adolescents (10.4 ± 2.5 years) with BSCP received a frNMS intervention targeting the gluteal muscles (12 sessions within 3 weeks). **Results**: In 77.1% of the sessions, no side effects were reported. In 16.7%, 6.3% and 5.2% of the sessions, a tingling sensation, feelings of pressure/warmth/cold or very shortly lasting pain appeared, respectively. frNMS was highly accepted by families (100% adherence) and highly feasible (97.9% of treatment per training protocol). A total of 100% of participants would repeat frNMS, and 87.5% would recommend it. The Canadian Occupational Performance Measure demonstrated clinically important benefits for performance in 28% and satisfaction in 42% of mobility-related tasks evaluated by caregivers for at least one follow-up time point (6 days and 6 weeks post intervention). Two patients accomplished goal attainment for one mobility-related goal each. One patient experienced improvement for both predefined goals, and another participant experienced improvement in one and outreach of the other goal as assessed with the goal attainment scale. **Conclusions**: frNMS is a safe and well-accepted neuromodulatory approach that could improve the quality of life, especially in regard to activity and participation, of children and adolescents with BSCP. Larger-scaled studies are needed to further explore the effects of frNMS in this setting.

## 1. Introduction

One of the most common neurological disorders in children is cerebral palsy [1] due to congenital or early acquired brain injury with a prevalence of 2.11 per 1000 births [1,2]. Next to the predominant motor disability, seizures, are frequent, and impairment in sensation, cognition and perception, language and communication, and behavior are often diagnosed [3]. The majority of children with CP have a spastic subtype, leading to multi-level contractures if not adequately managed on a long-term basis [4]. In addition to spasticity, weakness as well as impaired selective motor control represent important clinical key features of bilateral spastic CP (BSCP) within the pathophysiological framework of the upper motor neuron syndrome [5]. Multi-modal treatment is essential to support participation and quality of life [6,7,8,9].

Within the last few years, addressing weakness and impaired selective motor control has become a therapy goal as equally important as managing spasticity. Patients with BSCP often walk slowly with constrained or excessive joint movements (e.g., flexed knees or adducted and internally rotated hips), which is more exhaustive [10,11,12]. These gait deviations often deteriorate over time and contribute to the development of crouch gait, a pattern with excessively flexed hips and knees, affecting 74–88% of patients with BSCP [13,14,15]. Crouch gait is exacerbated by flexor contracture and extensor weakness of the lower extremities, including the gluteal muscle group. In children with BSCP, hip abductor strength accounts for a substantial degree of the variance in walking speed, gross motor function and mobility [16,17]. The requirements on these intrinsically weak muscles (in particular, on the gluteal muscles) to accelerate the center of mass forward and upward are especially high through crouch gait [18]. Next to the adverse effects on the lower extremity, abductor insufficiency affects pelvic and trunk movement, resulting in the Trendelenburg sign (pelvic drop to opposite side), Duchenne deviations (excessive trunk lean towards the ipsilateral leg) or a combination of both [19,20,21,22]. Strengthening the hip abductors and extensor may, thus, be an effective possibility to prevent the development of biomechanical malalignments of the lower extremity, decrease compensatory movements (trunk lean), facilitate ambulation and contribute to better dynamic balance.

In addition to orthoses and aids to support standing and walking, conventional and instrumented physiotherapy (e.g., partial body weight support or robot-assisted treadmill training, whole-body vibration training) represents an important approach to enhance power and endurance in children with BSCP [6]. However, if a child is not capable of selectively controlling a distinct muscle or muscle group, the efficacy and sustainability of these treatments might be limited. To overcome these boundaries, sensorimotor processing during motor training might be strengthened with external stimulation. This ideally results in a higher efficiency and sustainability of physical or occupational training. Repetitive neuromuscular magnetic stimulation (rNMS)—previously commonly referred to as repetitive peripheral magnetic stimulation (rPMS)—in combination with a task-specific motor training represents a neurostimulating approach that could deliver such external stimuli. Specifically, rNMS is based on the principle of electromagnetic induction and is non-invasive [23,24,25]. A copper-winded coil serves as a magnetic field generator and is attached to a stimulator, which generates an electric current. The hereby-induced magnetic field passes through the skin and, in turn, generates an electrical current of physiological dimension within the tissue [26,27]. This provokes muscle contractions by the direct stimulation of terminal motor branches [28]. In addition to a training effect at the muscular level, rNMS can also increase proprioceptive afferent information indirectly by activating the muscle spindles and mechanoreceptors of the muscle–tendon unit, joints and the skin. In addition, the terminal afferent nerve branches in joint capsules, ligaments and the skin are likely to be directly depolarized [23,24,29]. By these mechanisms of action, sensorimotor processing is modulated at the corticospinal as well as the cortico-cortical level within the framework of a neuromodulation from bottom up [23,24,25,30].

Against this background, we hypothesized that a protocol, developed by our research group, of a functional rNMS (frNMS) training applied to the gluteal muscles of children and adolescents with BSCP might be beneficial. Here, data on its safety and feasibility, in terms of adherence, practicability and satisfaction, as well as preliminary data regarding the clinical effects from the patients’ and their caregivers’ perspectives are presented.

## 2. Materials and Methods

### 2.1. Ethics

The institutional review board of the medical faculty approved this monocentric, prospective, uncontrolled, open-label clinical study (vote 20-604, ethical approval date: 18 August 2020). This study was conducted in accordance with the Declaration of Helsinki and was registered at the German Registry for Clinical Studies. Informed written consent of the participants and their caregivers was a prerequisite for study participation.

### 2.2. Study Design

Patients with BSCP, who are seen in our outpatient clinic on a regular basis, were offered study participation if they fulfilled the following criteria: diagnosis of BSCP, Gross Motor Function Classification System (GMFCS) Level I to III, age between 6 years and 17 years and 11 months and insufficient hip extension during standing and/or walking (definition of children in our study: 6 to 11 years and 11 months, adolescents 12 years to 17 years and 11 months). The exclusion criteria included general contraindications for magnetic stimulation (e.g., epilepsy, ferromagnetic implants, implanted biomedical devices including shunt systems), intellectual disability (IQ < 70), confirmed attention deficit (hyperactivity) disorder, orthopedic surgery or injection of botulinumtoxin to the lower limbs within the previous three months and a hip flexion contracture >15°. If the family was interested in study participation, an frNMS trial session was scheduled. In the case of opting for taking part in the study afterwards, the following appointments were a priori scheduled: Baseline assessment followed by the first frNMS training session within a maximum of 6 days; altogether 12 frNMS training sessions within 3 weeks; a short-term follow up assessment (FU) within a maximum of 6 days after the last frNMS training and a long term-follow up assessment at the timepoint of 6 weeks (FU-6) after the last frNMS training.

### 2.3. frNMS Intervention

**Figure 1:**
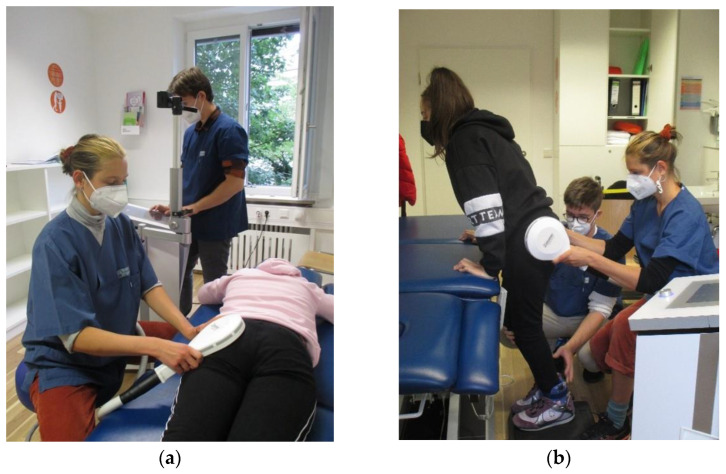
(**a**) static stimulation of the gluteal muscles as a “warm-up”; (**b**) stimulation of the left gluteal muscles while bringing the right leg up.

**Figure 2:**
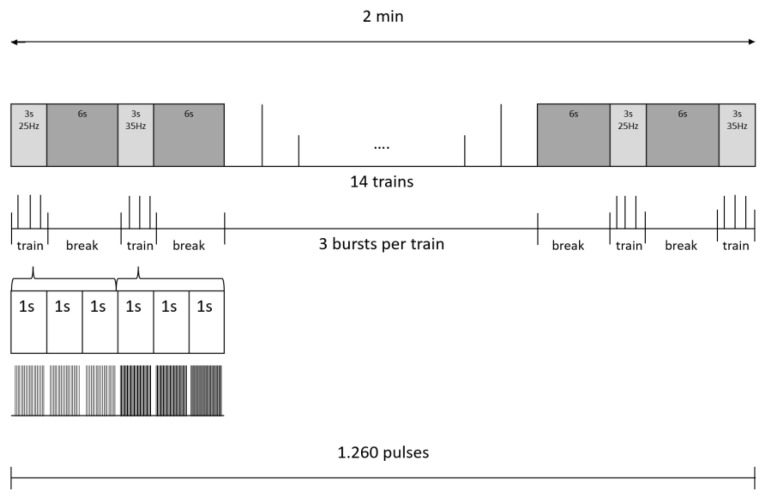
Stimulation protocol for the frNMS intervention targeting the gluteal muscles.

A board-certified physiotherapist supervised all frNMS sessions, which were performed by therapists who were thoroughly trained in the application of frNMS: Every treatment session aimed for 20 min of net stimulation time (10 min per body side). The stimulator (emField Pro, Zimmer MedizinSysteme GmbH, Neu-Ulm, Germany) was equipped with a self-cooling round coil with a diameter of the copper winding of 12.5 cm and a maximum output of 3 Tesla. The stimulator emitted rectangular-shaped, single pulses of 412 µs with the direction of the induced current from the outside to the inside of the coil. This stimulation coil was held in hand by the therapist in a position that assured a distinct contraction of the gluteal muscles (Figure 1). During the active exercises, the therapist continuously followed the movements of the participant to assure an effective stimulation throughout. The ON-time for stimulation was set to 3 s, and the OFF-time was set to 6 s, while the frequency alternated between 25 Hz and 35 Hz, respectively, summing up to a maximum of 12,600 stimuli emitted during 20 min of frNMS training. These parameters were already preset in a customized software program (Figure 2). The intensity of the stimuli was adapted by the therapists at an individual level for the relaxed baseline position of each exercise and body side. The intensity was slowly increased in steps of 6 to 10%, starting at 20% maximum output until a pronounced muscle contraction of the gluteal muscles was clearly visible without voluntary activation by the patient and without causing any pain or discomfort.

Each treatment session started with a “warm-up” that consisted of 2 min of static stimulation of the gluteal muscles (see Figure 1). Then, we combined the rNMS with physical exercises targeting the gluteal muscles during On-time (hip extension, abduction or external rotation; Supplementary Table S1). One therapy session included 5 exercises, which were repeated for 2 min on both sides each, once the adequate stimulation intensity had been defined. All 21 predefined exercises were designed to adhere to the concept of physiotherapy to promote motor learning, and these exercises focused on real-life motor tasks and activities [6,31]. The exercises were chosen by the physiotherapist together with the participant to meet the preset treatment goal, and they were adjusted to the individual gross motor capabilities of each participant (e.g., by using a foam pad when doing squats for very high-performing children).

### 2.4. Safety

The patients and therapists completed customized questionnaires after every session to document and assess any adverse events (AE) that had been experienced during the session. Prior to the start of each session, the participants and caregivers were asked to report any AEs experienced between sessions (Supplementary S1).

### 2.5. Feasibility

The definition of adherence was completing a minimum of 11 of the 12 scheduled sessions. For practicability, the therapists documented the performed exercises, level of difficulty and number of repetitions during all sessions. The number of ON-periods needed to define the appropriate stimulation spot and intensity together with the finally set intensity were also documented for each exercise on each body side. The therapists were asked to document any adaption of the preset stimulation and training protocol. Further notes during and after the treatment sessions informed about the highlights and challenges during the frNMS training as well as suggestions for improvement. To assess the overall satisfaction with the treatment, customized questionnaires (semi-structured and open comment options) were completed after every second session by the participants as well as at the end of the intervention by the participants and caregivers (Supplementary S2).

### 2.6. Patient- and Caregiver-Reported Effects

Using the Canadian Occupational Performance Measure (COPM) interview, 0–4 (by participants) and 2–8 (by caregivers) individual performance issues related to mobility were identified. These tasks were rated with regard to the level of performance and satisfaction with performance on a scale from 1 to 10 (with a score of 1 representing the lowest level of performance/no satisfaction at all and 10 meaning very-well performed/extremely satisfied) [32]. The COPM interview was held at baseline prior to the frNMS intervention, and changes over time were reassessed at follow-up at 6 days (short-term) and 6 weeks after the last frNMS session (long-term) together with the same interviewer without reference to the baseline scoring (blinded scoring). Based on the available literature, the COPM cutoff values for clinically important changes were set to 1.37 for performance and 1.9 for satisfaction [33].

The Goal Attainment Scale (GAS) was completed to describe the patients’ and caregivers’ treatment goals [34,35]. For each patient, two individual goals were defined at baseline prior to the frNMS intervention, and progress was rated on a 5-point scale at the short- and long-term follow-up (0 = achievement of predefined goal; +1/+2 = small/substantial improvement beyond defined goal; −1 = the patient has improved and progressed towards the defined goal but has not achieved it; −2 = the patient’s status has not changed, no improvement/aggravation) [36].

### 2.7. Data Management

The patient characteristics, details of the frNMS sessions, COPM as well as GAS scores were documented using paper-based clinical report forms, and questionnaires were filled in using paper forms. All data were entered into Microsoft Excel spreadsheets (Microsoft Office Professional Plus 2016, version 16.78, Microsoft, Redmond, WA, USA). The cross-checking of data entry was performed by at least two independent analysts.

### 2.8. Statistical Analysis

All statistical analyses were performed using Microsoft Excel (Microsoft Office Professional Plus 2016, version 16.78, Microsoft, Redmond, WA, USA) and SPSS (version 27; IBM SPSS Statistics for Windows, Armonk, NY, USA). The absolute and relative frequencies, means, standard deviations (SDs), medians and ranges were calculated for the subject and intervention characteristics, AEs and reports of satisfaction.

AEs were analyzed based on their absolute and relative frequencies. Completion of the intervention was defined as having attended a minimum of 11 of the 12 sessions. The percentage of patients having completed the frNMS intervention was taken as the adherence rate. Practicability was descriptively explored on behalf of the adherence to the stimulation and training protocol and the therapists’ comments. The motivation to undergo the frNMS intervention again and recommend it was used to assess the satisfaction based on the absolute and relative frequencies as well as the classification of the overall evaluation of the intervention.

The COPM and GAS datasets were tested for normal distribution with Shapiro–Wilk tests and, thereafter, for statistically significant changes from baseline to the short- or long-term follow-up with the appropriate tests: normally distributed COPM scores with paired *t*-tests and not normally distributed GAS scores with Wilcoxon signed-rank tests; statistical results are presented within the respective tables. The level of statistical significance was set at *p* < 0.05.

## 3. Results

### 3.1. Study Population

Screening for study eligibility of children and adolescents with BSCP was performed in the institution’s outpatient clinic. The eligible patients and their caregivers were educated about the frNMS intervention and possibility to participate in the study. If the family was willing to participate, a frNMS trial session was scheduled. Of the 34 eligible patients, 15 were educated about the frNMS intervention and the possibility to participate in the study. The remaining 19 families were not contacted since the patients currently or recently had received another therapeutic intervention (e.g., robot-assisted treadmill training). Seven of the contacted families denied a training session due to concurrent therapies, and/or limited time resources, and/or too long travel time to the clinic. All eight families, whose child underwent a trial session, consecutively opted for study participation (five females, mean age at baseline: 10 years and 4 months, SD 2 years and 5 months; Table 1).

### 3.2. Safety

No serious adverse events occurred. In 74 of the 96 sessions (77.1%), no AEs were reported. A tingling sensation within the stimulated body region was experienced by one patient in 10 sessions, and by two patients in all together six sessions, summing up to 16 reports (16.7% of sessions). During six sessions (6.3%), feelings of pressure, warmth or cold at the stimulated region were recorded. Pain was reported altogether five times (5.2%) by two patients during the intervention, only lasting for a few seconds. In between sessions, muscle soreness was recorded after two sessions (2.1%), tingling in the fingers after two sessions (2.1%) and a feeling of weakness (not to objectify by neurological examination) after one session by one patient each. In addition to the repositioning of the stimulation coil for AEs occurring during the stimulation, no further steps had to be taken regarding any AEs. None of the AEs led to a discontinuation of the therapy session or the end of the study participation.

### 3.3. Feasibility

The adherence rate was 100%, as all patients completed all planned sessions (12 sessions). Each session lasted approximately 45 to 60 min, depending on factors like motivation and attention as well as the level of demand of the chosen exercises. It took, on average, 3.1 ON-phases (SD 1.4) to determine the appropriate intensity for each exercise and body side. The mean stimulation intensity of all exercises performed by all patients yielded 48% of the maximum stimulator output (SD 37.5%) with a range of 10 to 100%. In one session of two participants, respectively, only four instead of five exercises were trained. During the remaining 94 sessions (97.9%), all five exercises with all aimed at repetitions were performed. The 10 most frequently performed physical exercises are listed in Table 2. Directly after a session, the treatment was rated as a positive experience in 97.9%. All patients and caregivers would repeat the intervention, and 87.5% of the patients as well as 75% of the caregivers would recommend frNMS to other children with BSCP. Additional comments regarding the intervention given by the participants and caregivers in the free-text boxes of the questionnaires are compiled in Supplementary Table S2.

### 3.4. Patient- and Caregiver-Reported Effects

The patient-reported COPM change from baseline to the short-term follow-up translated into a clinically important increase regarding the performance of one mobility-related task in four participants (overall 4/19 tasks) and a decrease in performance ratings in, altogether, 5/19 tasks (*p* > 0.05; Table 3a). Satisfaction improved for at least one task in four participants (overall 8/19 tasks) and decreased in, altogether, 5/19 tasks (*p* > 0.05). The change was sustained until the long-term follow-up for four tasks for performance (two increased, two decreased ratings) and six tasks for satisfaction (four increased, two decreased ratings), given the available reports of four participants at that time point (*p* > 0.05). One participant experienced a clinically meaningful improvement in performance and satisfaction regarding an additional task during the time span from the short- to long-term follow-up, whereas one participant reported a decline in satisfaction for one task. Regarding all 36 mobility-related tasks evaluated by the caregivers at the short-term follow-up, performance/satisfaction improved in 6/10 and declined in 4/3 (*p* > 0.05). Based on 28 tasks evaluated at the long-term follow-up, performance/satisfaction improved in 7/7 and declined in 2/3 compared to the baseline ratings (*p* = 0.025 performance; *p* > 0.05 satisfaction) (Table 3b).

Concerning GAS, improvement for both pre-defined goals were reported for one participant and goal attainment for one goal in two other participants, respectively. Another participant experienced improvement towards one goal and outreached the other goal. Overall, the short-term improvement was significant (*p* = 0.014). All improvements were sustained at the long-term follow-up (*p* = 0.017; Table 4).

## 4. Discussion

This study reports the first experience regarding the safety of and feasibility by the means of adherence to, practicability of and satisfaction with a personalized frNMS intervention addressing the gluteal muscles offered to children and adolescents with BSCP, GMFCS Level II and III. In addition, the important perspective of the patients and their caregivers, regarding preliminary clinical effects of the intervention, is presented as well.

In line with previous reports of rNMS as a treatment of other neurological conditions, frNMS demonstrated an excellent safety profile with a low rate of AEs [24,25,29,37,38,39]. None of the AEs demanded for a change or discontinuation of the treatment. frNMS was very well-accepted. The patients and their caregivers were very satisfied with the intervention, translating into a high motivation to repeat and recommend frNMS. Overall, the intervention turned out to be very feasible, as the treatment was conducted according to the protocol in 97.9% of the sessions.

To date, two publications reported on rNMS interventions in six patients aged 6 to 11 years with BSCP, GMFCS level I and II [40,41]. The treatment comprised five sessions of static rNMS, applying 1800 stimuli during each session. The targets of stimulation were the tibial and peroneal nerve to induce a contraction in the ankle plantar- and dorsiflexors [40,41]. Yet, the current study investigated a protocol of magnetic stimulation applied simultaneously during physical exercises. This functional approach was chosen because evidence supports dynamic motor training to be superior to static settings [6]. In addition, the gluteal muscles represented the stimulation target for the first time in this study. By this choice, frNMS aimed at an increased stability and endurance during standing, walking and climbing stairs, as well as an ease of transfer in-between positions by fostering strength and muscular activation as demanded by the participants and their caregivers. Accordingly, the concrete physical exercises and their level of demand were chosen to meet these goals, depending on the patients’ priorities. Thus, this frNMS concept addressed meaningful activities of daily living as well as participation in a highly personalized way.

Regarding the COPM, substantial improvements were reported by the participants/caregivers in this study for 26%/28% of mobility-related problems regarding the level of performance and for 47%/42% regarding the level of satisfaction for at least one follow-up time point. Subjective functioning was rated as decreased regarding performance in 26%/11% and regarding satisfaction in 32%/14%. The individually tailored selection of exercises for each participant as well as the different levels of attention during the training and attitudes towards the intervention may contribute to the differences in the patient-reported clinical outcomes. The age of the children might play a role with regard to responsiveness as well. Patients 5 and 6, who are adolescents (12 years and older), both reported improvements for performance and satisfaction for at least one mobility-related task for both follow-up time points, whereas in the group of children (patients 1–4, 7 and 8), only two reported an improvement for one or two mobility-related tasks at the follow-up time point one for performance, and two patients reported improvements of satisfaction at time point one and/or two (but only reports of four children were available). In the caregivers’ COPM reports, benefits were also pronounced in the adolescent group. Whether better comprehension of the frNMS intervention together with a potentially better cooperation in the adolescents is a reason for this finding cannot be answered at this time.

Emphasizing the patients’ and/or caregivers’ aims and progress towards them, the patient-reported outcome measures represent an important aspect of this study. The results of such assessments will help to identify mobility-related problems that will respond better to the treatment. This will help to stratify patients to different types of interventions in the future, depending on their personal mobility-related goals. Another strength is the 6-week follow-up period to explore the sustainability of the primarily observed effects. As in other studies, the caregivers tended to underestimate progress compared to the children [42]; we chose to take into account both perspectives. Although the predefined goals included many tasks demanding complex mobility, improvements were experienced and observed on the individual level.

Although frNMS is associated with a high demand of personal resources with two therapists involved in each session, this approach still seems highly suitable for children and adolescents for several reasons. Next to the very good safety profile, rNMS normally does not cause any pain or discomfort, even at high intensity levels [23,24,25]. Since the magnetic field penetrates tissues of all kinds without being considerably attenuated, there is no need for attaching electrodes or taking clothes off in comparison to transcutaneous electrical stimulation. Further, rNMS reaches even more profound muscles and triggers a more pronounced muscle contraction than transcutaneous electrical stimulation [24,29,43].

So far, eight patients were treated in this study. The sample size limits generalizability regarding the presented findings, in particular with regard to the preliminary patient/caregiver reported outcomes. To further assess the benefits of frNMS, future controlled studies are warranted, either implementing a sham-controlled, a conventional physiotherapy “only” or instrumented training group (e.g., partial body weight support or robot-assisted treadmill training, whole-body vibration). Biomechanical assessment of gait and isometric muscle strength via motion capturing or dynamometric technology may also objectively quantify whether the desired positive effects of improved gluteal strength have been achieved and transfer to ambulation. In addition, protocols assessing neurophysiological outcomes (e.g., corticospinal excitability with transcranial magnetic stimulation; cerebral perfusion, volume and integrity of tracts, as well as network connectivity with advanced neuroimaging) may inform about the distinct mechanisms of action and may be appropriate to evaluate dose–response interactions. Based on such dose–response curves, the most adequate stimulation protocol could be chosen for future trials. Objective endpoints together with outcome measurements evaluating the patients’ and caregivers’ perspectives in a larger sample size are needed to assess the distinct effects of this frNMS intervention in children and adolescents. The excellent safety and feasibility profile demonstrated in the current study paves the way for such randomized controlled trials.

## 5. Conclusions

The results of this clinical study might support the use of frNMS in children and adolescents with BSCP to train the gluteal muscles. The safety and feasibility of the frNMS protocol were excellent. The individual participant- and caregiver-reported improvements should encourage larger-scaled, controlled studies to further investigate the neurophysiological background of frNMS and the potential for clinically meaningful improvements regarding activity and participation as well as function in children and adolescents with BSCP.

## Figures and Tables

**Table 1:** Characteristics of the participants undergoing the frNMS intervention. Abbreviations: BoNT Botulinum toxin; BW (g) birth weight (in grams); F female; GA (w + d) gestational age (in weeks and days); GMFCS Gross Motor Function Classification System; M male; m months; MRI magnetic resonance imaging; PVL periventricular leukomalacia; y years; * age at baseline assessment.

Patient	Sex	Age *	GMFCS Level	MRI	GA (w + d)	BW (g)
1	M	10 y 7 m	III	PVL	32 + 3	1820
2	F	8 y 11 m	II	PVL	28 + 5	1040
3	M	10 y 3 m	II	MRI without corresponding finding	41 + 4	3930
4	F	6 y 11 m	II	PVL	29 + 3	1140
5	F	13 y 2 m	III	PVL	29 + 1	1355
6	F	14 y 3 m	III	PVL	26 + 2	730
7	F	11 y 11 m	III	PVL	33 + 0	2150
8	M	7 y 1 m	II	PVL	40 + 0	2975

**Table 2:** Top 10 most frequently selected exercises for frNMS targeting the gluteal muscles in children with BSCP.

Nr.	Position	Focus	Description	Performed in n Sessions
1	Standing, squatting position	Extension	Straightening up when stimulated	36
2	Standing	Abduction	Abducting a leg when stimulated	35
3	One-leg stand	Extension	Bringing one leg up when stimulated	30
4	Prone position	Extension	When stimulated, both legs go up while the rest of the body stays on the ground	29
5	Supine position, flexed arms	Extension	Bringing the hip up when stimulated	28
6	Quadruped position	Extension	“Kicking” back with the active leg when stimulated	26
7	Standing	Extension	Kicking a ball when stimulated	26
8	Standing	Extension	Taking a step up when the other leg is stimulated	23
9	Standing	Extension	Maintaining balance on wobbly ground	23
10	Lateral position	External rotation	Rotating the active leg outwards/upwards with heels touching each other at all times	21

**Table 3:** (**a**): COPM reported by participants. Participants 2 and 8 were not able to define goals by themselves; ** not reported as patient underwent percutaneous myofasciotomy during interval; *** participant herself was not available for long-term follow-up. (**b**): COPM reported by caregivers. ** not reported as patient underwent percutaneous myofasciotomy during interval; **** not able to rate. (**a**,**b**) GMFCS Gross Motor Function Classification Level; BL baseline; FU short-term follow-up; FU-6 follow-up after 6 weeks; bold/italic printed = improvement/decrease of >1.37 for performance and >1.9 for satisfaction compared to BL [33].

**(a)**									
**Domain**				**Performance**			**Satisfaction**		
**Patient**	**GMFCS Level**	**Goal**		**BL**	**FU**	**FU-6**	**BL**	**FU**	**FU-6**
1	III	Overall		5	4.3	3.8	5.3	3.8	3.5
		Task 1	Walking without assistance (150 m)	2	2	1	2	1	1
		Task 2	Walking upstairs with support of one hand	5	**7**	5	5	*3*	*3*
		Task 3	Walking downstairs with support of one hand	5	*3*	*3*	4	3	*2*
		Task 4	Walking without assistance (dining table to couch)	8	*5*	*6*	10	*8*	*8*
2	II	--	--	--	--	--	--	--	--
3	II	Overall		5	**7**	6	7	**9**	**9**
		Task 1	Walking without upper body swaying	5	**7**	6	7	**9**	**9**
4	II	Overall		2	3	**	1	**6**	**
		Task 1	One-leg-jump l.	2	3	**	1	**6**	**
		Task 2	One-leg-jump r.	2	3	**	1	**6**	**
5	III	Overall		4.8	6	6	3.8	5.3	5
		Task 1	Leg streching while walking	5	6	6	3	**5**	4
		Task 2	Hold balance when standing up	5	6	6	4	**6**	5
		Task 3	Stability while standing	3	**6**	**5**	3	**5**	**5**
		Task 4	Walking endurance	6	6	7	5	5	6
6	III	Overall		2.7	3.7	**4.7**	1.3	**3.7**	**4.3**
		Task 1	Walking endurance	4	5	5	2	**5**	**4**
		Task 2	Walking upstairs without railing	3	*1*	**5**	1	1	**5**
		Task 3	Jumping far with both legs	1	**5**	**4**	1	**5**	**4**
7	III	Overall		6.2	*4.6*	***	6.4	4.8	***
		Task 1	Standing up with help	7	6	***	7	6	***
		Task 2	Walking effortlessly with walker and orthosis	8	7	***	8	7	***
		Task 3	Walking endurance with walker and orthosis	6	5	***	6	*4*	***
		Task 4	Walking effortlessly with help of another person	5	*3*	***	6	*4*	***
		Task 5	Standing free with orthosis	5	*2*	***	5	*3*	--
8	II	--	--	--	--	--	--	--	--
Mean (SD)				4.6 (2.0)	4.6 (1.9)	5.1 (1.1)	4.3 (1.9)	4.8 (2.1)	5.5 (2.4)
BL to FU				*p* = 0.909			*p* = 0.318		
BL to FU-6				*p* = 0.223			*p* = 0.231		
**(b)**									
**Domain**				**Performance**			**Satisfaction**		
**Patient**	**GMFCS Level**	**Goal**		**BL**	**FU**	**FU-6**	**BL**	**FU**	**FU-6**
1	III	Overall		3.3	3.3	**5**	3	3.3	**6**
		Task 1	Walking without assistance (dining table to couch)	1	2	**5**	1	2	**8**
		Task 2	Walking without assistance (hallway to railing)	5	**7**	**9**	5	**7**	**9**
		Task 3	Toiletting independently	4	*1*	*1*	3	*1*	*1*
2	II	Overall		5.5	5.2	5.2	5.5	5.8	5
		Task 1	Straight posture	5	5	5	5	6	6
		Task 2	Enduring stable standing on both legs	5	6	6	5	6	5
		Task 3	Walking endurance without assistance	6	6	6	6	6	6
		Task 4	Leg streching while walking	6	*4*	*4*	6	6	5
		Task 5	Stop from walking	5	5	5	5	6	4
		Task 6	Turn around on the spot while walking	6	5	5	6	5	*4*
3	II	Overall		5.5	6	6	5.5	7	6
		Task 1	Stability of upper body while walking	6	6	6	6	7	6
		Task 2	Stability of legs while walking	5	6	6	5	**7**	6
4	II	Overall		4.8	4.6	**	3.6	4.6	**
		Task 1	Walking upstairs without railing	2	2	**	2	2	**
		Task 2	Walking downstairs without railing	2	2	**	2	2	**
		Task 3	Stability while standing (when being pushed)	5	5	**	5	5	**
		Task 4	Keep left heel down when walking (with orthosis)	5	4	**	3	4	**
		Task 5	Keep left heel down when walking (without orthosis)	6	5	**	3	**5**	**
		Task 6	Walking endurance with orthosis	6	7	**	4	**7**	**
		Task 7	Walking endurance without orthosis	7	7	**	5	**7**	**
		Task 8	Keep left foot on pedal while riding a bike	5	5	**	5	5	**
5	III	Overall		3.3	**6.3**	3.5	4.3	**7**	3.8
		Task 1	Leg streching while walking	3	**7**	3	4	**7**	4
		Task 2	Hold balance when standing up	5	****	4	5	****	4
		Task 3	Stability while standing	3	**6**	3	4	**7**	3
		Task 4	Walking endurance	4	**6**	4	5	**7**	4
6	III	Overall		5	5.3	**7.7**	5.7	5.3	**7.7**
		Task 1	Walking endurance	3	**7**	**6**	4	**6**	**6**
		Task 2	Balance while standing	6	*3*	**9**	6	*3*	**9**
		Task 3	Balance while walking	6	7	**8**	6	7	**8**
7	III	Overall		6	5.2	6.8	6.4	4.8	7.8
		Task 1	Standing up with help	6	6	**9**	6	5	**9**
		Task 2	Walking effortlessly with walker and orthosis	8	*6*	8	8	7	9
		Task 3	Walking endurance with walker and orthosis	7	6	8	7	7	**9**
		Task 4	Walking effortlessly with help of another person	4	3	4	4	3	4
		Task 5	Standing free with orthosis	5	5	5	7	*2*	8
8	II	Overall		2.2	3	3.6	3.4	3	3
		Task 1	Walking endurance	6	6	6	5	5	5
		Task 2	Stability while walking	2	2	5	5	1	5
		Task 3	Walking with heels on the ground	1	1	1	3	2	*1*
		Task 4	Sitting on the ground without help of arms	1	**5**	**4**	3	**6**	3
		Task 5	Riding a bike with training wheels	1	1	2	1	1	1
Mean (SD)				4.5 (1.8)	4.8 (1.9)	5.3 (2.2)	4.6 (1.6)	4.9 (2.1)	5.4 (2.5)
BL to FU				*p* = 0.378			*p* = 0.292		
BL to FU-6				*p* = **0.025**			*p* = 0.140		

**Table 4:** Achievement with regard to goals pre-defined and reported with GAS. GMFCS Gross Motor Function Classification Level; BL baseline; FU short-term follow-up; FU-6 follow-up after 6 weeks; * not observed during interval; ** not reported as patient underwent percutaneous myofasciotomy during interval *** not able to rate; for participant 8, only one goal had been defined a priori.

Goal Nr.		1			2		
Patient Nr.	GMFCS Level	BL	FU	FU-6	BL	FU	FU-6
**1**	III	Free walking inside the flat with orthesis without help of a carer			Stairs down with adjusting step; one hand on the railing		
		−2	−1	−1	−2	−1	−1
**2**	II	Straight posture with knees extended			Walking endurance measured by steps taken without pausing		
		−2	−2	−2	−2	−1	0
**3**	II	Riding a bike without training wheels			Stability when walking without upper body swaying		
		−2	−2	*	−2	−2	−2
**4**	II	Walking endurance with orthesis (up to the park)			Stairs up and down without a carer		
		−2	−2	**	−2	−2	**
**5**	III	Straight posture while walking			Climb stairs without a carer/railing		
		−2	***	−2	−2	***	−2
**6**	III	Walking endurance			Dress up (put on trousers) faster		
		−2	0	0	−2	−2	−2
**7**	III	Walking endurance (with support/orthoses)			Stability when standing free with orthoses		
		−2	0	1	−2	−1	−1
**8**	II	Riding a bike equipped with training wheels			--		
		−2	−2	−2	--	--	--
Mean (SD)		−2 (0)	−1.3 (0.9)	−1.0 (1.2)	−2 (0)	−1.5 (0.5)	−1.3 (0.7)
BL vs. FU		*p* = **0.014**					
BL vs. FU-6		*p* = **0.017**					

## Data Availability

The data presented in this study are available upon request from the corresponding author. The data are not publicly available due to the sensitive character of pediatric clinical data.

## Supplementary Materials

The following supporting information can be accessed online: https://www.mdpi.com/article/10.3390/children10111768/s1, Table S1: frNMS targeting to the gluteal muscles. Predefined physiotherapeutic exercises; of that, a set was chosen for the intervention according to the goals and capabilities of the individual participant; Table S2: frNMS targeting to the gluteal muscles. Free-text feedback of participants and their caregivers given in the questionnaires during and after the intervention; S1: Treatment documentation questionnaires completed prior and after every session assess any adverse events occurring during or after treatment session; S2: Questionnaires for participants and their caregivers used to assess the satisfaction with the frNMS treatment.

## Abbreviations

AE	adverse events
BSCP	bilateral spastic cerebral palsy
COPM	Canadian Occupational Performance Measure
CP	Cerebral palsy
frNMS	functional repetitive neuromuscular magnetic stimulation
GAS	Goal Attainment Scale
GMFCS	Gross Motor Function Classification System
rNMS	repetitive neuromuscular magnetic stimulation
rPMS	peripheral magnetic stimulation
SDs	standard deviations
