# Supplementary material for: Safety and Feasibility of Functional Repetitive Neuromuscular Magnetic Stimulation of the Gluteal Muscles in Children and Adolescents with Bilateral Spastic Cerebral Palsy
Source: Children (Basel). 2023 Oct 31;10(11):1768. doi: 10.3390/children10111768 (PMC10670153; doi:10.3390/children10111768)
Supplement: Supplementary file 1 [file children-10-01768-s001.zip › 220907_supplemental S1_rnms gluteus_feasbility.pdf]

Supplemental S1: Treatment documentation questionnaire, completed prior and after every session assess any adverse events occurring during or after treatment session

frNMS Treatment Documentation

Study-ID: ______________________

Session Nr.: ____________

Date: __________________________

1. Did the last rNMS session cause any adverse events? If so, …

… which did you notice?

- ☐ Pain
- ☐ Feelings of Pressure
- ☐ Muscle Sore
- ☐ Muscle Tremor
- ☐ Tingle
- ☐ Numbness
- ☐ Cold Feelings
- ☐ Warm Feelings
- ☐ Burning Sensation
- ☐ Furry Feelings
- ☐ Other Adverse Events: ____

- ☐ No

… when did they occur?

- ☐ In Motion
- ☐ While Resting
- ☐ Constantly/Always

… how strong where they (VAS 0-10)? __________________________________

… how long did they last? ____

2. Did the patient feel pain in hip or legs before the beginning of the session?

- ☐ No
- ☐ Yes

3. Did the patient report any adverse events or unpleasant sensations during the rNMS session? If so, …
… which occured?

- ☐ Pain
- ☐ Feelings of Pressure
- ☐ Muscle Sore
- ☐ Muscle Tremor
- ☐ Tingle
- ☐ Numbness
- ☐ Cold Feelings
- ☐ Warm Feelings
- ☐ Burning Sensation
- ☐ Furry Feelings
- ☐ Other Adverse Events: ____

- ☐ No
