# Supplementary material for: Safety and Feasibility of Functional Repetitive Neuromuscular Magnetic Stimulation of the Gluteal Muscles in Children and Adolescents with Bilateral Spastic Cerebral Palsy
Source: Children (Basel). 2023 Oct 31;10(11):1768. doi: 10.3390/children10111768 (PMC10670153; doi:10.3390/children10111768)
Supplement: Supplementary file 1 [file children-10-01768-s001.zip › 220907_supplemental S2_rnms glutes_feasibility.pdf]

Supplemental S2: Questionnaires for participants and their caregivers used to assess the satisfaction with the frNMS treatment

Patient's questionnaire after every second session

1. This questionnaire was filled in by:

    ☐ me

    ☐ my parent/a third person

2. This questionnaire was filled in on: ______________________

3. Were you positioned comfortably during the session?

☐ Yes

☐ No, because ____

____

4. How did the treatment feel?

____

____

5. Did you experience any pain or discomfort during the session?

☐ Yes; please specifiy the severity of the pain using the emoticons below and let us know, where you experienced pain:

____

____

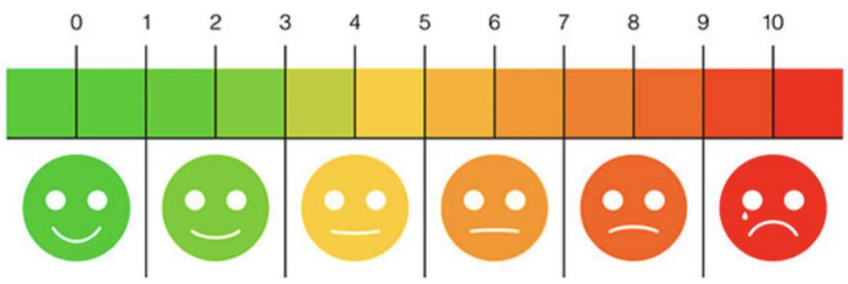

☐ No

6. Would you repeat this treatment?

☐ Yes

☐ No, because… ____

____

7. Does the treated body part feel different now?

☐ Yes, how?

____

____

☐ No

8. Does any other body part feel different now (sensation of heat, itching, burning)?

☐ Yes, where? ____

____

☐ No

9. Further comments:

____

____

____

____

Thank you very much for answering these questions and for your feedback!

Questionnaire for patients after the treatment with rNMS

Study-ID: ____________________________________

1. This questionnaire was filled in by:

☐ me ☐ caregiver/a third person

2. This questionnaire was filled in on: ____________________

3. Overall, how did you experience the treatment?

____

____

4. Would you undergo the treatment again?

☐ Yes

☐ No, because ____

____

5. Does the treated body region feel different in comparison to before the intervention?

☐ Yes, how?

____

____

☐ No

6. Does any other body region feel different in comparison to before the intervention?

☐ Yes, where?

____

____

☐ No

7. Would you recommend this treatment method to other children/adolescents with movement restrictions?

☐ Yes

☐ No, because ____

____

8. Could/Should we have done anything differently during the treatment?

☐ Yes, what?

____

____

☐ No

10. Further comments:

____

____

____

____

Thank you for completing the questionnaire!

Questionnaire for caregivers at the end of the patients´ rNMS treatment

Study-ID: ____

1. This questionnaire was filled in by: ☐ Mother ☐ Father
2. This questionnaire was filled in on: ____________________
3. Do you notice a difference in your childs behavior in comparision to before the treatment?

   ☐ No
   ☐ Yes, which?

   ____

   ____

4. Did your child comment on how it liked the treatment?
   ☐ No
   ☐ Yes, how?

   ____

   ____

5. Did your child complain about pain or other sufferings (headache, pain in legs, bottom or hip etc.)?

   ☐ No
   ☐ Yes, where and what?

   ____

   ____

6. Would you let your child undergo the treatment again?
   ☐ Yes
   ☐ No, because ____

7. Would you recommend this therapy to other familys with children with movement restrictions?

☐ Yes
☐ No, because____

8. Do you have any ideas to improve the process?
☐ No

☐ Yes, which?

Further comments:

9. Further comments:

Thank you for completing the questionnaire!
