# Supplementary material for: Safety and Feasibility of Functional Repetitive Neuromuscular Magnetic Stimulation of the Gluteal Muscles in Children and Adolescents with Bilateral Spastic Cerebral Palsy
Source: Children (Basel). 2023 Oct 31;10(11):1768. doi: 10.3390/children10111768 (PMC10670153; doi:10.3390/children10111768)
Supplement: Supplementary file 1 [file children-10-01768-s001.zip › 220907_supplemental table S1_rnms gluteus_feasibility_excercises.pdf]

Suppl. Table S1: frNMS targeting to the gluteal muscles. Predefined physiotherapeutic exercises, of that a set was chosen for the intervention according to the goals and capabilities of the individual participant

Nr.	Position	Focus	Execution	Stimulated Leg	
0	Prone position	---	Static stimulation as “Warm-Up”	Both	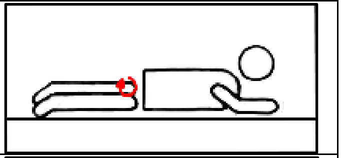
1	Supine position, flexed arms, knees bent	Extension	Bringing the hip up when stimulated	Both	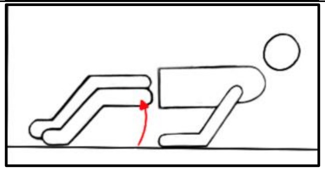
2	Sitting position, straight arms	Extension	Bringing the hip up when stimulated	Both	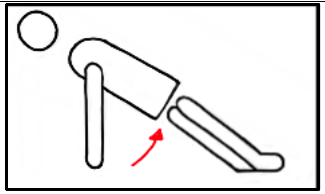
3	Side position, knees bent	External rotation	Outwards rotation of the upper side knee while feet stay together	Active leg	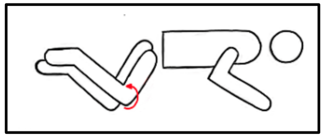
4	4-point position	Extension	“Kicking” back with the active leg when stimulated	Active leg	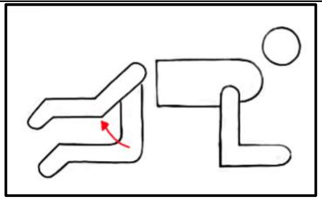

5	Prone position	Extension	When stimulated, both legs go up while the rest of the body stays on the ground	Both	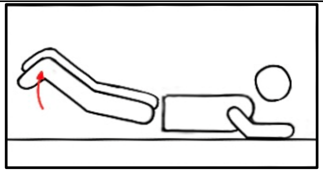
6	4-point position	External rotation	Rotating the active leg outwards/upwards when stimulated	Active leg	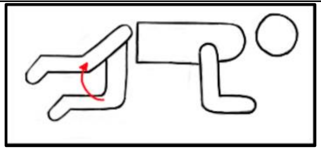
7	Standing	Extension	Kicking a ball when stimulated	Both	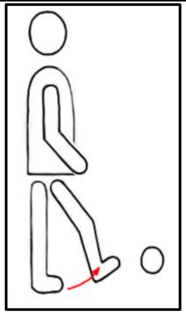
8	Standing	Abduction	Abducing a leg when stimulated	Both	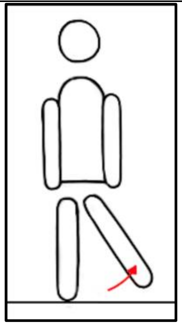

9	Standing	Extension	When stimulated, the active leg slides back behind the passive leg	Active leg	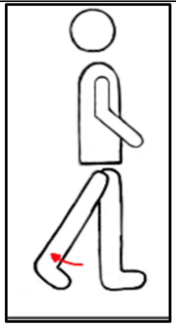
10	Standing, legs shifted, front leg bent	Extension	Extension of the front leg when stimulated	Active leg	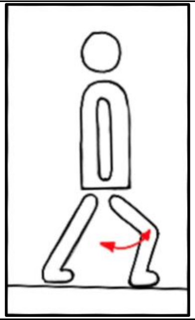
11	Half-kneeling position	External rotation	Stimulation of the standing leg	Standing leg	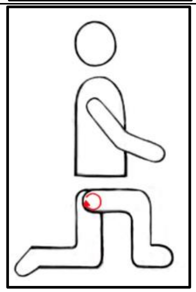

12	Standing	Extension	Taking a step up when the other leg is stimulated	Passive leg	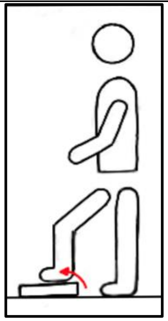
13	Standing, squatting position	Extension	Straightening up when stimulated	Both	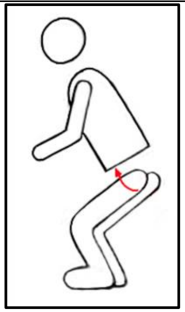
14	Standing	Extension	Maintaining balance on wobbly ground	Both	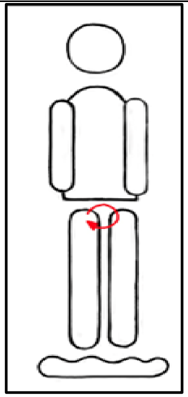

15	One-leg-stand	Extension	Bringing one leg up when stimulated	Passive leg	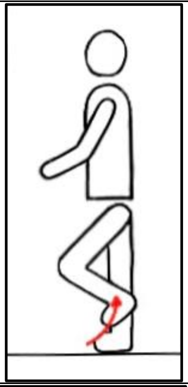
16	Sitting on elevated position	Extension	Standing up when stimulated	Both	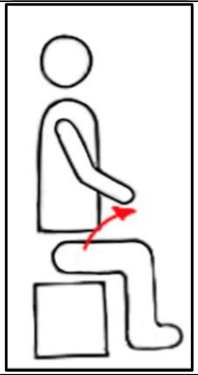
17	Kneeling position	External rotation, Abduction	From kneeling to half-kneeling position when stimulated	Both	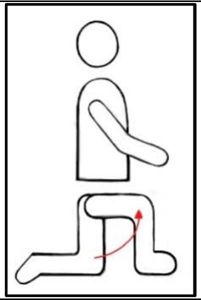

18	Standing, upper body laying on elevated position	Extension	When stimulated, the active leg slides behind the passive leg	Active leg	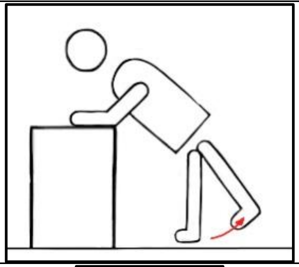
19	Kneeling position	Extension	From sitting kneeling position to standing kneeling position when stimulated	Both	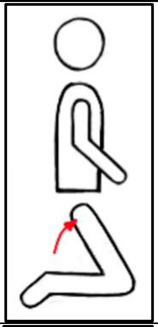
20	Standing	External rotation		Active leg	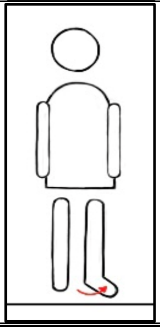
21	Prone position, one leg bent upwards	External rotation	The bent leg “falls” onto the straight leg	Active leg	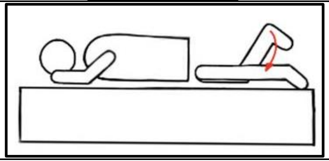
