# Supplementary material for: Safety and Feasibility of Functional Repetitive Neuromuscular Magnetic Stimulation of the Gluteal Muscles in Children and Adolescents with Bilateral Spastic Cerebral Palsy
Source: Children (Basel). 2023 Oct 31;10(11):1768. doi: 10.3390/children10111768 (PMC10670153; doi:10.3390/children10111768)
Supplement: Supplementary file 1 [file children-10-01768-s001.zip › 221116_supplemental table S2_rnms gluteus_feasibility_feedback_update.pdf]

Supplemental table S2: frNMS targeting to the gluteal muscles. Free-text feedback of participants and their caregivers given in the questionnaires during and after the intervention.

Participant	Time Point	Comment
**Participant 1:**		
Child	FU	“The therapy was challenging”
Caregiver	FU	“He now tries to stand free by himself without holding our hands, he is more confident”
	FU-6	“The stimulation had a visible effect, for a certain time span you could notice a strong improvement of his capabilities. However, after 3 months the effects declined again. I’m sure that in combination with physiotherapy or as an intensive, frequent treatment block, this treatment method will show effects“
**Participant 3:**		
Caregiver	FU	“(I noticed) a small improvement in terms of stability and a high motivation for the next session”<br><br>“(My child commented) only positively (on the intervention)”
**Participant 4:**		
Caregiver	FU	“She is more secure while climbing stairs, has less pain in her legs.”<br>“She asks more often, if she can walk by herself; she runs faster; she wants to be tickled at her foot sole”
**Participant 5:**		
Child	FU	“Interesting, it was an exciting time”
Caregiver	FU	“She is a little bit more stable”
	FU-6	“Our conclusion: it was a very good exercise, which had positive effects on strength and therefore our child’s stability, especially directly after the therapy sessions. However, we couldn’t notice a long-term improvement. Maybe a continuous intervention would have more effects”
**Participant 6:**		
Caregiver	FU	“She really liked the intervention; she was glad to come”
	FU-6	“She told me, that the gluteal muscles sometimes twitched exactly like they did during the stimulation until one week ago. She still loses her balance quite often.“
**Participant 7:**		
Child	FU	“The treatment felt good. I liked the exercises.”
Caregiver	FU	“She can walk better, is more stable and got better at climbing stairs.”
**Participant 8:**		
Child	FU	“I can run faster than before”
Caregiver	FU	“My child is more stable than before the treatment”
	FU-6	“He can now walk with his heels intentionally touching the ground. His kicks (football) are more powerful after the treatment.”
